# Supplementary material for: Perinatal serotonin signalling dynamically influences the development of cortical GABAergic circuits with consequences for lifelong sensory encoding
Source: Nat Commun. 2025 Jun 4;16:5203. doi: 10.1038/s41467-025-59659-5 (PMC12137630; doi:10.1038/s41467-025-59659-5)
Supplement: Supplementary file 1 — Supplementary Information [file 41467_2025_59659_MOESM1_ESM.pdf]

## Supplementary information

# Perinatal serotonin signalling dynamically influences the development of cortical GABAergic circuits with consequences for lifelong sensory encoding

Gabriel Ocana-Santero[1,2], Hannah Warming[1], Veronica Munday[1], Heather A. MacKay[1], Caius Gibeily[1], Christopher Hemingway[1], Jacqueline A. Stacey[1], Abhishek Saha[1], Ivan P. Lazarte[1], Anjali Bachetta[1], Fei Deng[3,4], Yulong Li[3,4], Adam M. Packer[1], Trevor Sharp[2], Simon. J. B. Butt[1,*]

* corresponding author: Simon Butt (simon.butt@dpag.ox.ac.uk)

Affiliations:

1. Dept. of Physiology, Anatomy and Genetics, Oxford University, Oxford, OX1 3PT, UK.
2. Dept. of Pharmacology, Oxford University, Oxford, OX1 3QT, UK
3. State Key Laboratory of Membrane Biology, Peking University School of Life Sciences, Beijing 100871, China.
4. PKU-IDG/McGovern Institute for Brain Research, Beijing 100871, China.

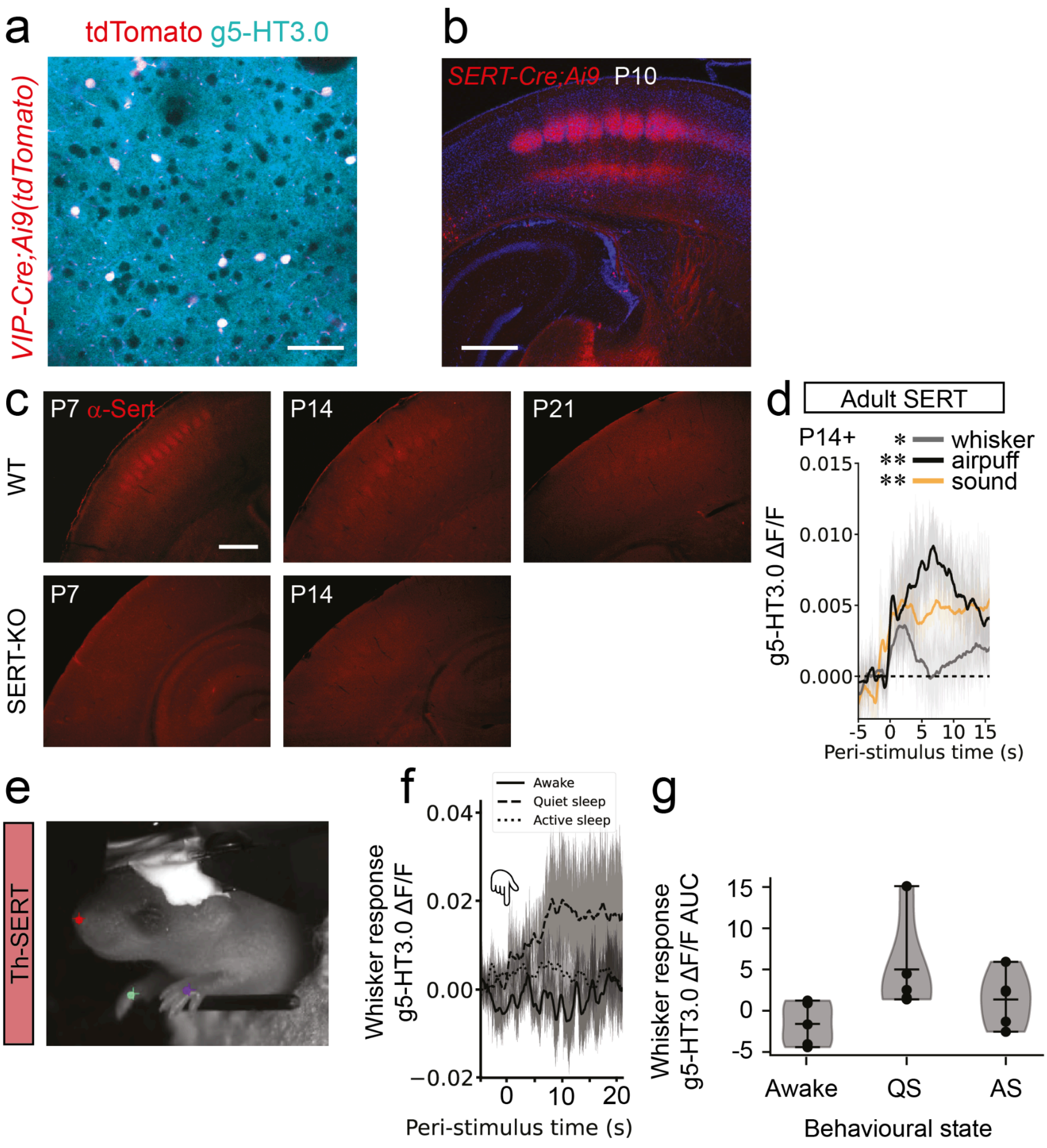


**Supplementary Fig. S1: Developmental serotonin dynamics. a,** S1BF layer 2/3 field of view in a P21 mouse expressing the genetically encoded fluorescent serotonin sensor g5-HT3.0 and tdTomato in VIP+ interneurons; scale bar: 50 µm. **b,** Confocal image of S1BF from a P10 transgenic *SERT-Cre;Ai9* animal conditionally expressing tdTomato in *SERT-Cre* neurons; scale bar: 405 µm. Fate-mapping neurons using *SERT-Cre* results in strong labelling of thalamocortical afferents in primary sensory areas due to transient SERT expression in first order thalamic nuclei during perinatal development [18]. **c,** Immunohisto-chemistry for SERT protein in wildtype (WT) and SERT knockout (SERT-KO) littermates across early postnatal development. Clear labelling of S1BF barrel cortex is present at P7 in WT animals only but this fades by P14/P21; scale bar: 325 µm. **d**, Average g5-HT3.0 signal in S1BF upon the presentation of 10 whisker (paired t-test p = 0.019), air puff (paired t-test p

= 0.006) or sound (paired t-test p = 0.009) stimuli in WT animals older than P14 (n=6). **e,** Frame from an infrared camera while 2-photon imaging was performed in a P8 pup. Manually labelled (circle) and deeplabcut[108] predicted (cross) markers overlap, showing tracking of nose (red), green (right forelimb) and purple (left forelimb), the latter used for behavioural state scoring. **f,** Average peri-stimulus g5-HT3.0 trace in response to whisker stimulation in P7-10 WT animals. **g**, g5-HT3.0 signal as a function of behavioural state (i.e., awake, quiet sleep (QS) or active sleep (AS)) and the corresponding areas under the curve (**g,** ANOVA sleep-state p = 0.07, n = 4).

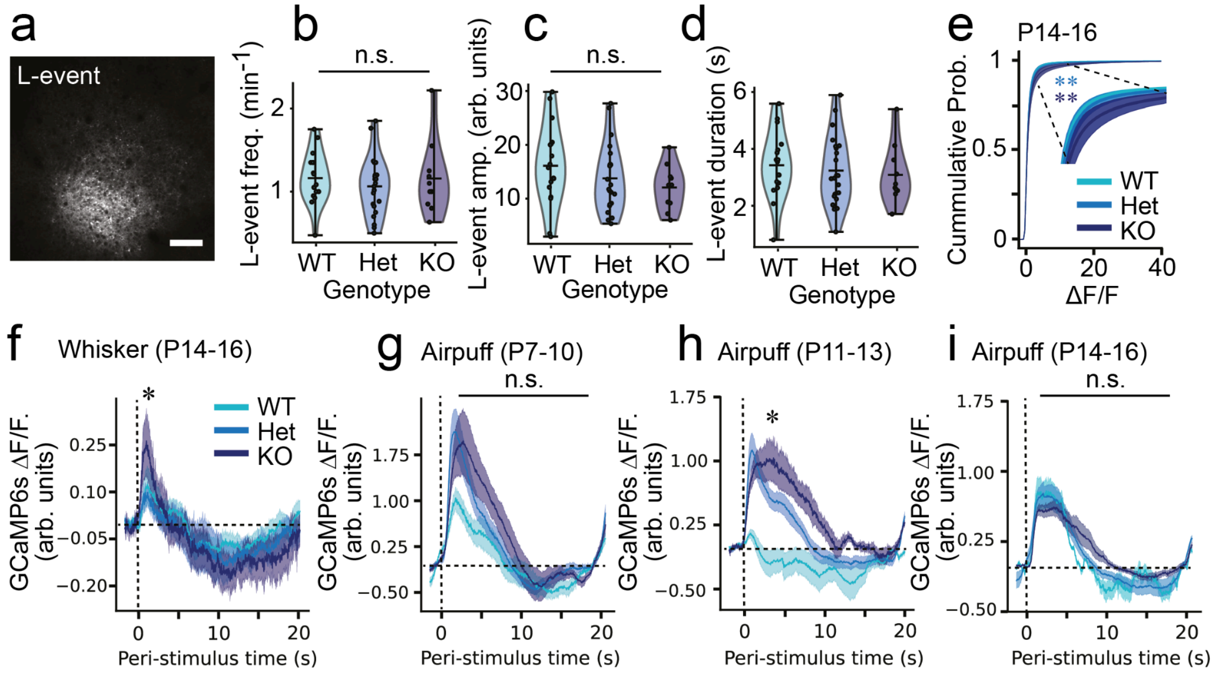


**Supplementary Fig. S2: SERT-KO transitions from cortical hypoactivity to hyperexcitability. a,** Representative field of view of a L-event, showing the characteristic neuronal recruitment of 20-80% of cells. Image made from an average of 120 frames (scale bar = 100 µm). **b,** Mean L-event frequency in SERT-WT/Het/KO mice at. (ANOVA genotype p = 0.074). **c,** Mean GCaMP6s signal amplitude of cells during L-events in SERT-WT/Het/KO mice (ANOVA genotype p = 0.062). **d,** Average L-event duration in SERT-WT/Het/KO mice (ANOVA genotype p = 0.563). **e,** Cumulative probability of ΔF/F 20 minute baseline values of all SERT-WT/Het/KO at P14-16 (Kolmogorov–Smirnov test: WT-HET $p < 0.001$; WT-KO $p < 0.001$). **f** Stimulus-triggered average GCaMP6s signal across all cells and mice showing increased whisker-response amplitude at P14-16 (ANOVA p = 0.025, pairwise WT-HET p = 0.464, WT-KO p = 0.030). **g,h,i** Stimulus-triggered average GCaMP6s signal across all cells and mice in response to an airpuff at P7-10 (**g,** ANOVA p = 0.670), P11-13 (**h,** ANOVA p = 0.047, pairwise WT-HET p = 0.198, WT-KO p = 0.038) and P14-16 (**i,** ANOVA p = 0.599). Sample sizes are 19 (WT), 23 (Het) and 10 (KO).

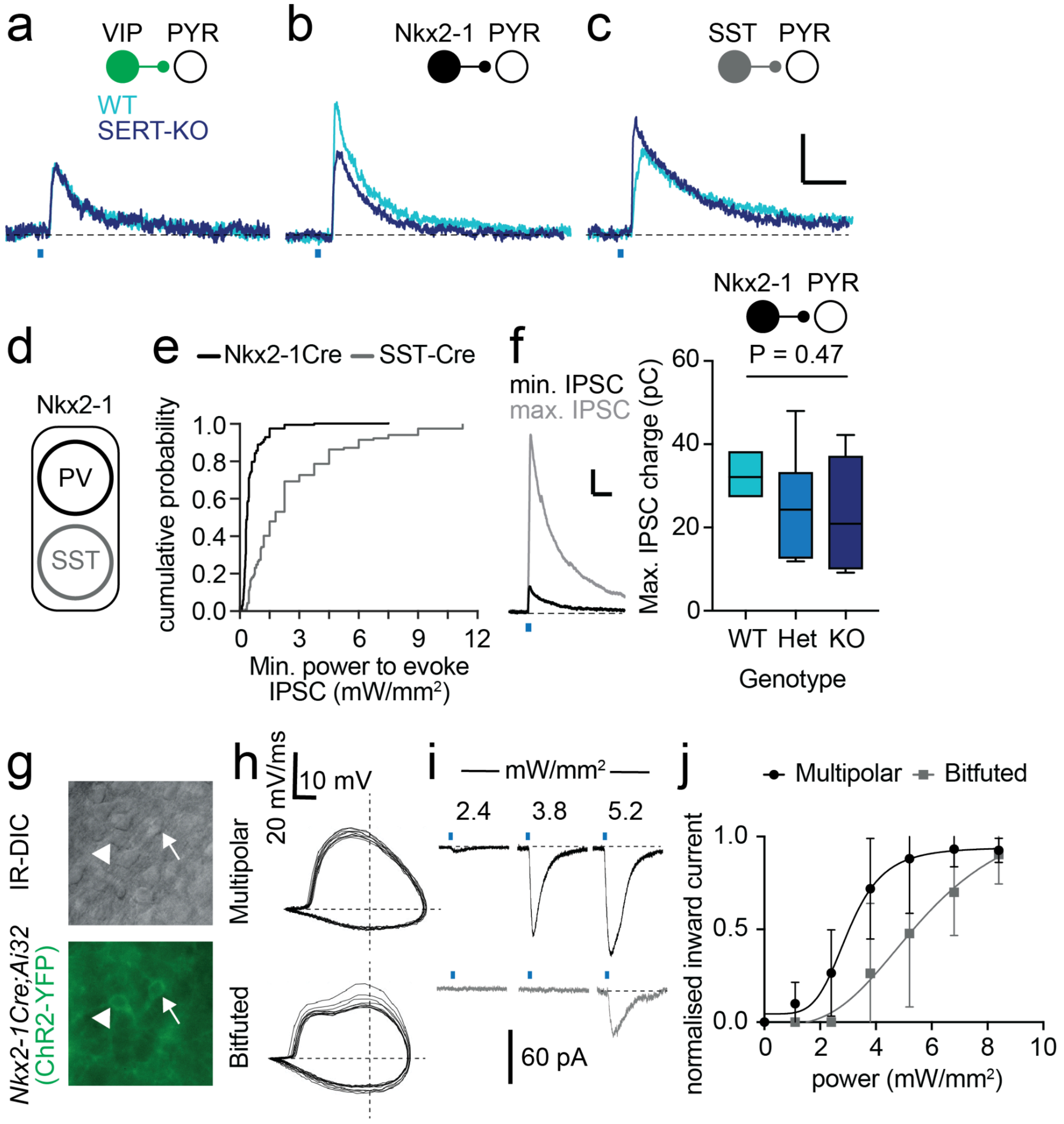


**Supplementary Fig. S3. *Ex vivo* characterization of changes in inhibitory microcircuitry of SERT-KO mice.** Representative superimposed traces of IPSCs elicited in L2/3 pyramidal cells following minimal ChR2-mediated stimulation of (**a**) VIP, (**b**) Nkx2-1 or (**c**) SST interneurons; WT (cyan) or SERT-KO (dark blue) mice. **d**, progenitors defined by expression of the transcription factor *Nkx2-1* give rise to primarily PV and SST expressing interneurons in the postnatal neocortex. **e**, Cumulative probability plot showing the minimal stimulation LED power used to evoke IPSC in PYRs recorded from both *Nkx2-1Cre* (black; n = 178 cells)) and *SST-Cre* (grey; n = 146) lines . IPSCs could be elicited at significantly less

power when using the *Nkx2-1Cre* line (P<0.0001; K-S test D:0.6304). **f**, (*left panel*) representative recording showing maximal (grey) stimulation IPSC superimposed on minimal stimulation IPSC recorded at $E_{Glut}$. *Right panel*, IPSC charge elicited by maximal stimulation of Nkx2-1 interneurons measured in PYRs across the different SERT genotypes (Anova F(2,21) = 0.8378;  P = 0.4466). **g**, (*top panel*) IR-DIC image of L2/3 neurons in an acute *ex vivo* slice of an *Nkx2-1Cre;Ai32* pup. *Bottom panel*, expression of YFP in multipolar (*white arrowhead*) and bitufted (*arrow*) interneurons in the same field of view. **h**, spike phase plot (dv/dt) for the two neurons shown in (**g**). **i**, representative inward currents elicited in (*top*; black traces) the multipolar, putative PV YFP+ neuron and (*bottom*; grey) equivalent from the bitufted, putative SST interneuron across a range of LED power (2.4 - 5.2 $mW/mm^2$). **j**, Plot of normalised inward current elicited by varying LED power in multipolar (n = 6 cells) and bitufted (n = 4) interneurons.

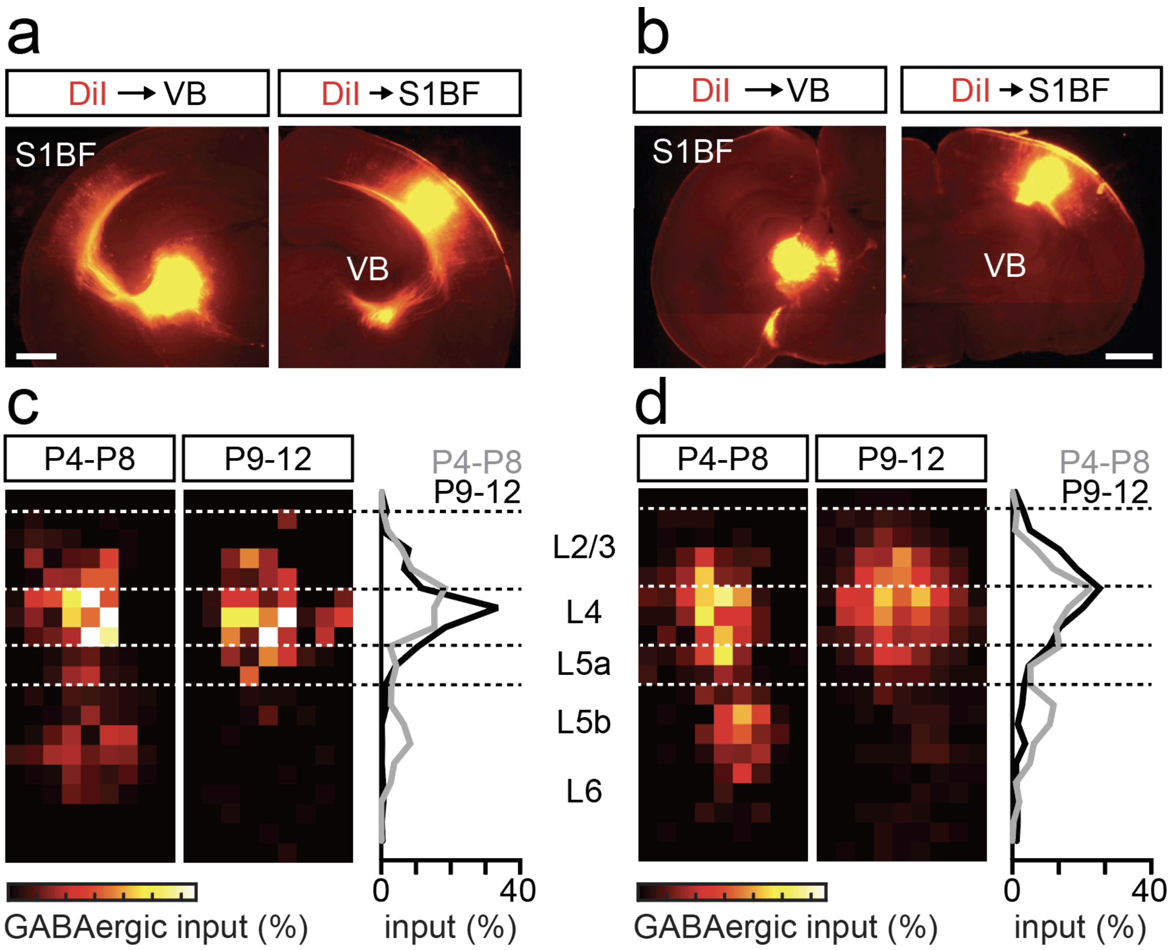


**Supplementary Fig. S4: Genetic deletion of thalamo-cortical and cortico-thalamic connectivity does not alter the timeline of the transient L5b GABAergic input onto L4 spiny stellate neurons (SSNs). a**, placement of DiI crystals in either ventrobasal (VB) thalamus (left) or S1BF (right) results in labelling of cortex and VB respectively in a P7 control (*Dlx5/6Cre;Celsr3+/-*) pup; scale bar: 600 μm. **b**, corresponding data for a P7 mutant (*Dlx5/6Cre;Celsr3-/-*) pup with no labelling of cortex (left) or thalamic nuclei (right). **c**, total GABAergic input onto L4 SSNs in control animals reveals early (P4-8), transient innervation by L5b interneurons (*left* map; n = 7 cells from 4 animals), that disappears post-L4 critical period plasticity (P9-12)(*middle plot*; n = 4 cells from 3 animals). *Right* panel, percentage GABAergic synaptic input onto L4 SSNs across the depth of S1BF. Dashed lines, average layer boundaries. **d**, Corresponding data for mutant animals lacking thalamocortical connectivity (P4-8: n = 12 cells from 7 animals; P9-12: n = 9 cells from 5 animals).

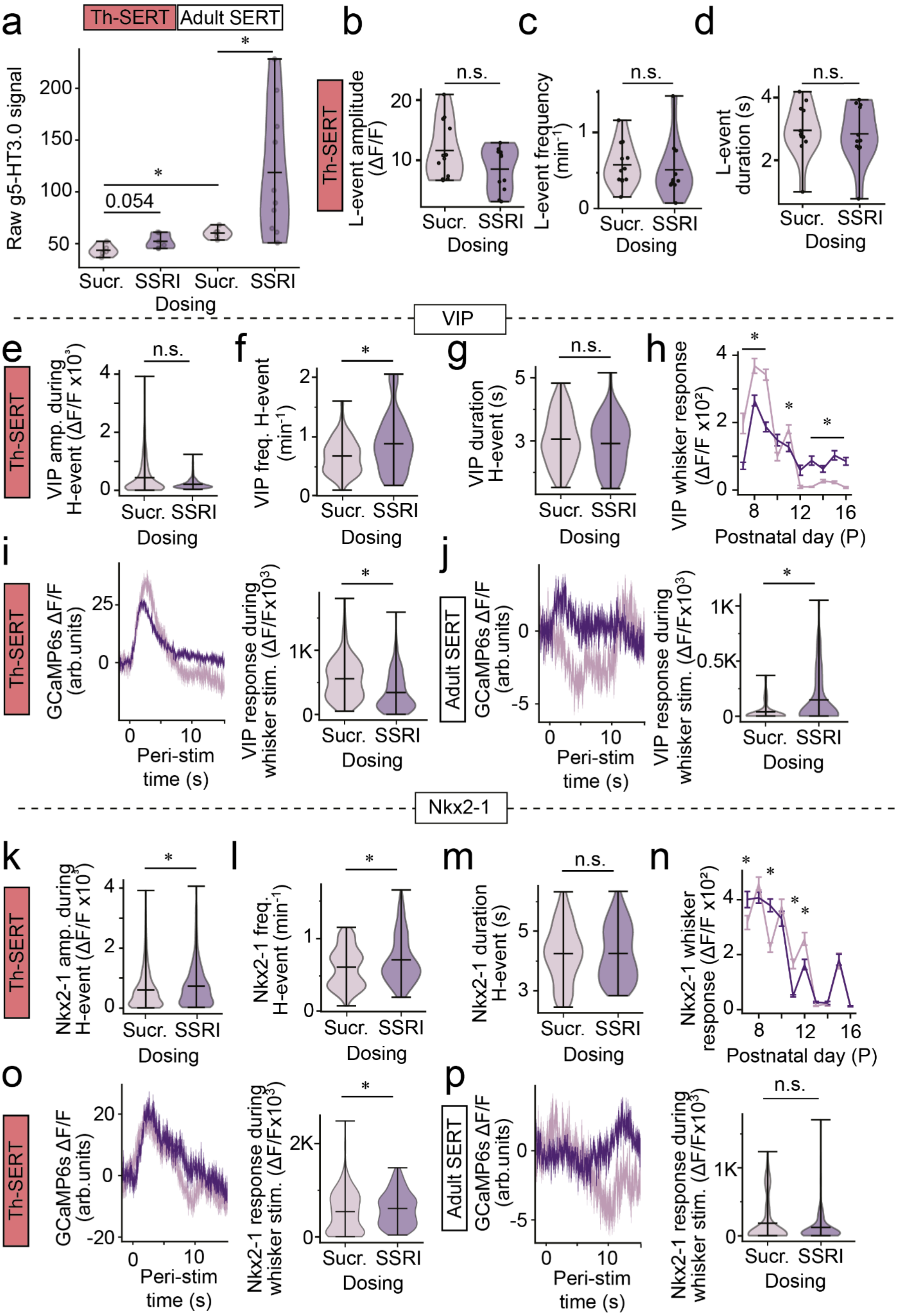
a
Th-SERT
Adult SERT
Raw g5-HT3.0 signal
0.054
Sucr.
SSRI
Dosing
b
Th-SERT
L-event amplitude (ΔF/F)
n.s.
c
L-event frequency (min-1)
d
L-event duration (s)
VIP
e
VIP amp. during H-event (ΔF/F x10³)
f
VIP freq. H-event (min-1)
g
VIP duration H-event (s)
h
VIP whisker response (ΔF/F x10²)
Postnatal day (P)
i
GCaMP6s ΔF/F (arb.units)
Peri-stim time (s)
VIP response during whisker stim. (ΔF/Fx10³)
j
Adult SERT
Nkx2-1
k
Nkx2-1 amp. during H-event (ΔF/F x10³)
l
Nkx2-1 freq. H-event (min-1)
m
Nkx2-1 duration H-event (s)
n
Nkx2-1 whisker response (ΔF/F x10²)
o
Nkx2-1 response during whisker stim. (ΔF/Fx10³)
p

**Supplementary Fig. S5: Postnatal SSRIs recreate SERT-KO early hypoactivity. a,** Mean raw g5-HT3.0 signal in Sucrose- or SSRI-postnatally-treated mice at Th-SERT and Adult SERT (Kruskal-Wallis test $p < 0.001$, Dunn pairwise comparison Th-SERT: Sucr.-SSRI $p = 0.054$; Adult SERT: Sucr.-SSRI $p = 0.039$; Sucr Th-SERT vs Sucr. Adult SERT $p = 0.0035$). Sample sizes from left to right are n = 5, n = 6, n = 5 and n = 10 mice. **b,c,d,** Mean L-event amplitude (t-test: $p = 0.098$), frequency (Mann–Whitney U: $p = 0.160$) and duration (t-test: $p = 0.774$) in Sucrose- or SSRI-treated mice at P7-10. Sample sizes are 12 (Control) and 12 (SSRI). **e,f,g** Violin plots of average amplitude (**e**), frequency (**f**) and duration (**g**) of VIP+ interneurons during H-events in Sucrose- or SSRI-treated mice at Th-SERT (**e,** Mann–Whitney U $p = 0.401$ **f** Mann–Whitney U $p < 0.001$, **g,** Mann–Whitney U $p = 0.366$). **h,** Whisker response amplitudes of VIP+ interneurons in Sucrose- and SSRI-treated mice from P8 to P16 (* $p < 0.05$). **i,j** VIP whisker response trace (left) and amplitude (right) of Sucrose- or SSRI-treated mice during the Th-SERT (**i,** Mann–Whitney U $p < 0.001$) and the Adult SERT period (**j,** Mann–Whitney U $p = 0.002$). **k,l,m** Violin plots of average amplitude (**k**), frequency (**l**) and duration (**m**) of Nkx2-1+ interneurons during H-events in Sucrose- and SSRI-treated mice at Th-SERT (**k,** Mann–Whitney U $p < 0.001$ **l** Mann–Whitney U $p = 0.006$, **m,** Mann–Whitney U $p = 0.881$). **n,** Whisker response amplitudes of Nkx2-1+ interneurons in Sucrose- and SSRI-treated mice from P8 to P16 (* $p < 0.05$). **o,p** Nkx2-1+ whisker response trace (left) and amplitude (right) of Sucrose- or SSRI-treated mice during the Th-SERT (**o,** Mann–Whitney U $p = 0.013$) and the Adult SERT period (**p,** Mann–Whitney U $p = 0.174$). VIP sample sizes are 870 cells from 5 animals (Control) and 576 cells from 6 animals (SSRI) (g-h), while Nkx2-1 are 1291 cells from 5 animals (Control) and 775 cells from 4 animals (SSRI) (i-j).

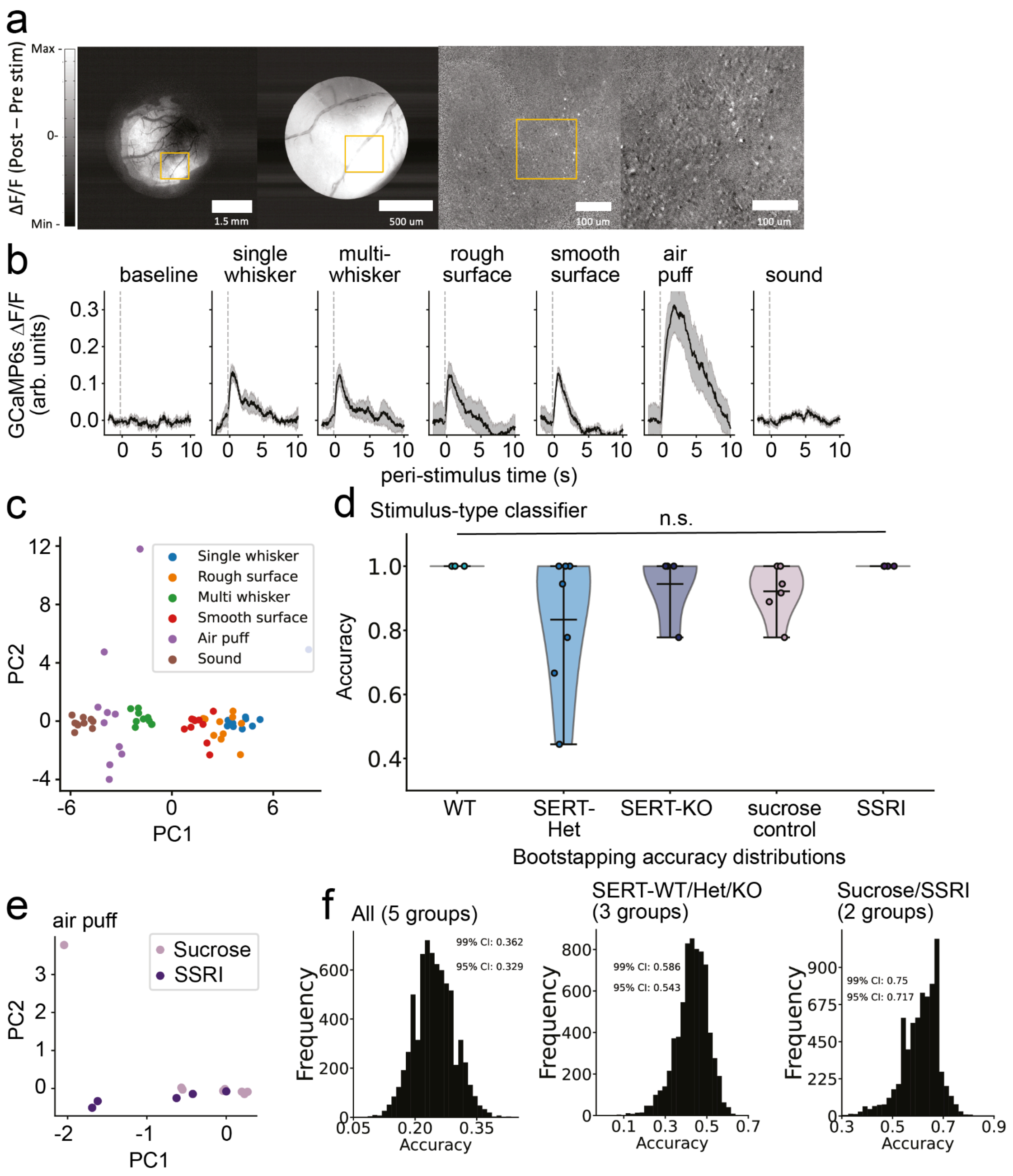


**Supplementary Fig. S6: Sensory encoding in adult somatosensory cortex of mice with developmental disruptions of SERT. a,** Example of the online analysis used to map single barrels: Stimulus-triggered average fluorescence post- minus pre- single whisker stimulation in 1-photon (left) or 2-photon (right) microscopy. Images are the average of 10 stimuli. **b,** Stimulus-triggered average calcium trace of 10 repeats of each stimulus type for a representative WT animal, from left to right: baseline, single-whisker, multi-whisker, rough surface, smooth surface, air puff and sound. **c,** Principal component analysis showing the spread of neuronal responses to the repeats of the different stimuli (colour-coded) along the two first components. Data from a single representative WT mouse. **d,** Testing classification accuracy of logistic regression classifiers for the decoding of the different types of stimulation

from the neuronal responses for WT, SERT-Het, SERT-KO, Sucrose-treated or SSRI-treated animals. A different classifier was trained for each animal. **e,** Principal component analysis showing the spread of neuronal responses to air puff stimulation along the two first components, for sucrose- and SSRI-treated animals. Each dot is a different animal. **f**, Null accuracy distributions generated by bootstrapping, i.e., training genotype/treatment classifiers on datasets with permuted labels. Sample sizes are 10 (WT), 8 (HET), 5 (KO), 6 (Control) and 5 (SSRI).

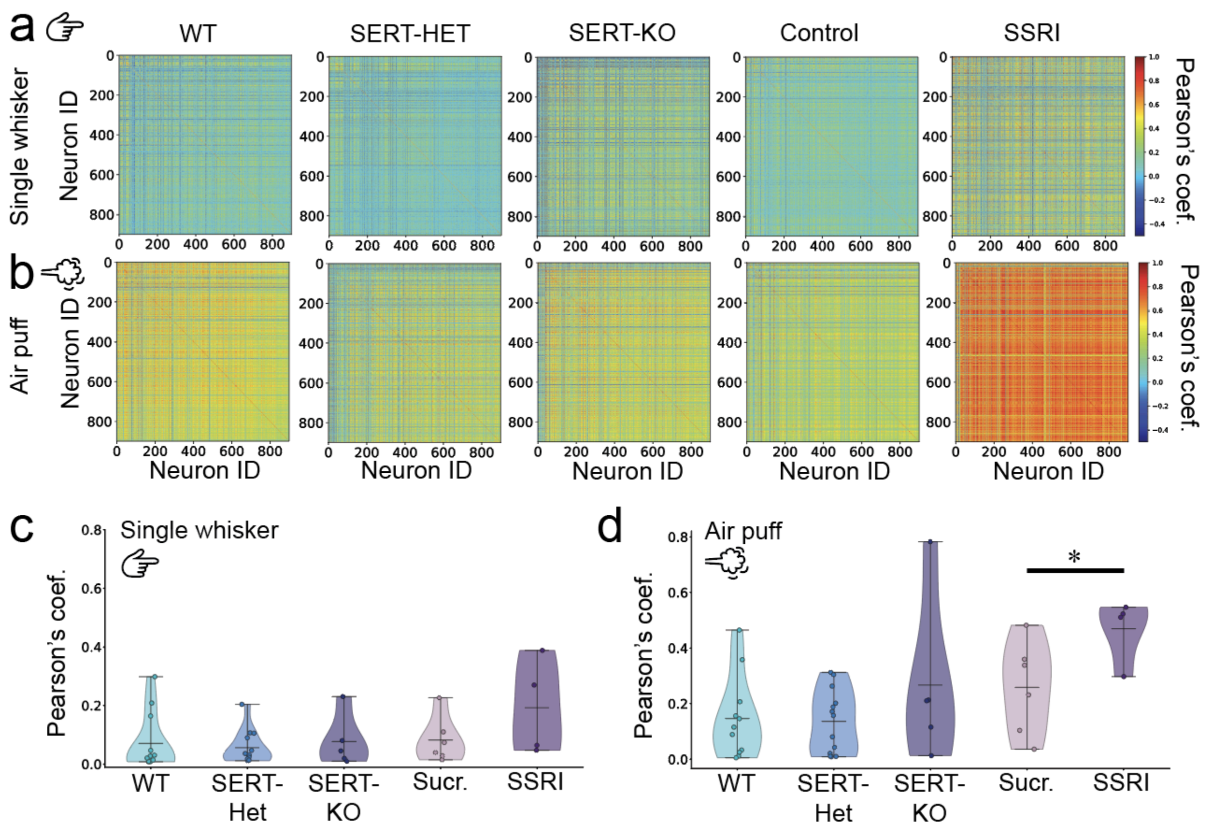


**Supplementary Fig. S7: Correlation analysis of single whisker and airpuff responses.**
**a,b** Representative heat maps showing Pearson's correlation coefficient across all cells in the field of view of WT, SERT-Het, SERT-KO, Sucrose and SSRI-treated mice during response to single whisker deflection (**a**) or air puff stimulation (**b**). **c,d,** Violin plots of average Pearson's correlation coefficient across the field of view during responses to single whisker deflection (**c,** Kruskal-Wallis test $p = 0.091$) and air puff stimulation (**d,** Kruskal-Wallis test $p = 0.016$, pairwise comparison Sucr-SSRI $p = 0.019$, WT-KO $p = 0.647$).

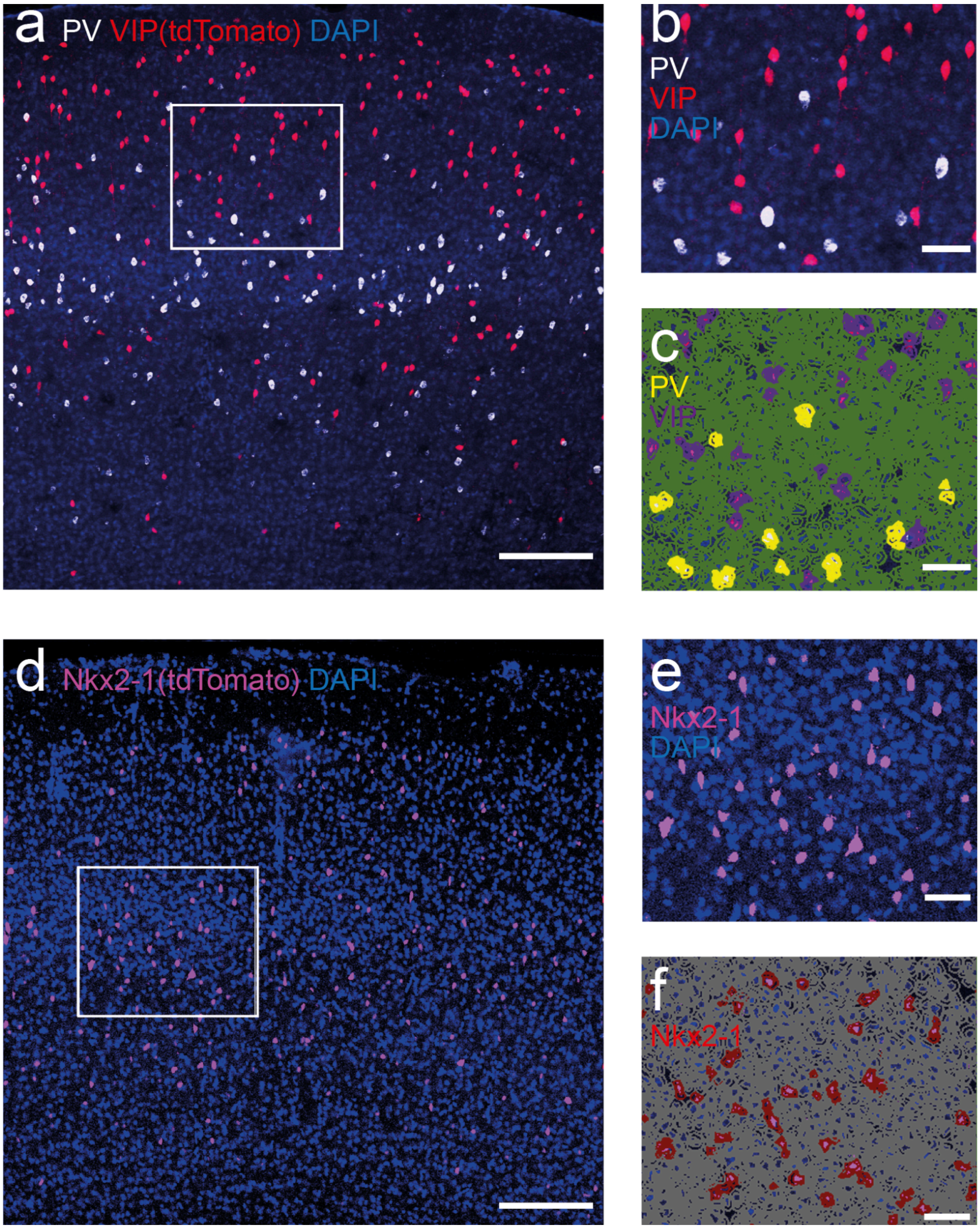


**Supplementary Fig. S8. Validation of automated interneuron classifiers. a,** Confocal image of S1BF on an adult VIP-tdTomato; scale bar: 200 um. **b,** Higher magnification image (scale bar: 50 um) of area in panel **a**, delineated by the white box with (**c**) comparisons to automated PV and VIP classifier detections. **d,** Confocal image of S1BF in an adult *Nkx2-1Cre;Ai9(tdTomato)* mouse. **e,** Same confocal image magnified ~2x, with comparisons to (**f**) automated Nkx2-1 classifier detections.

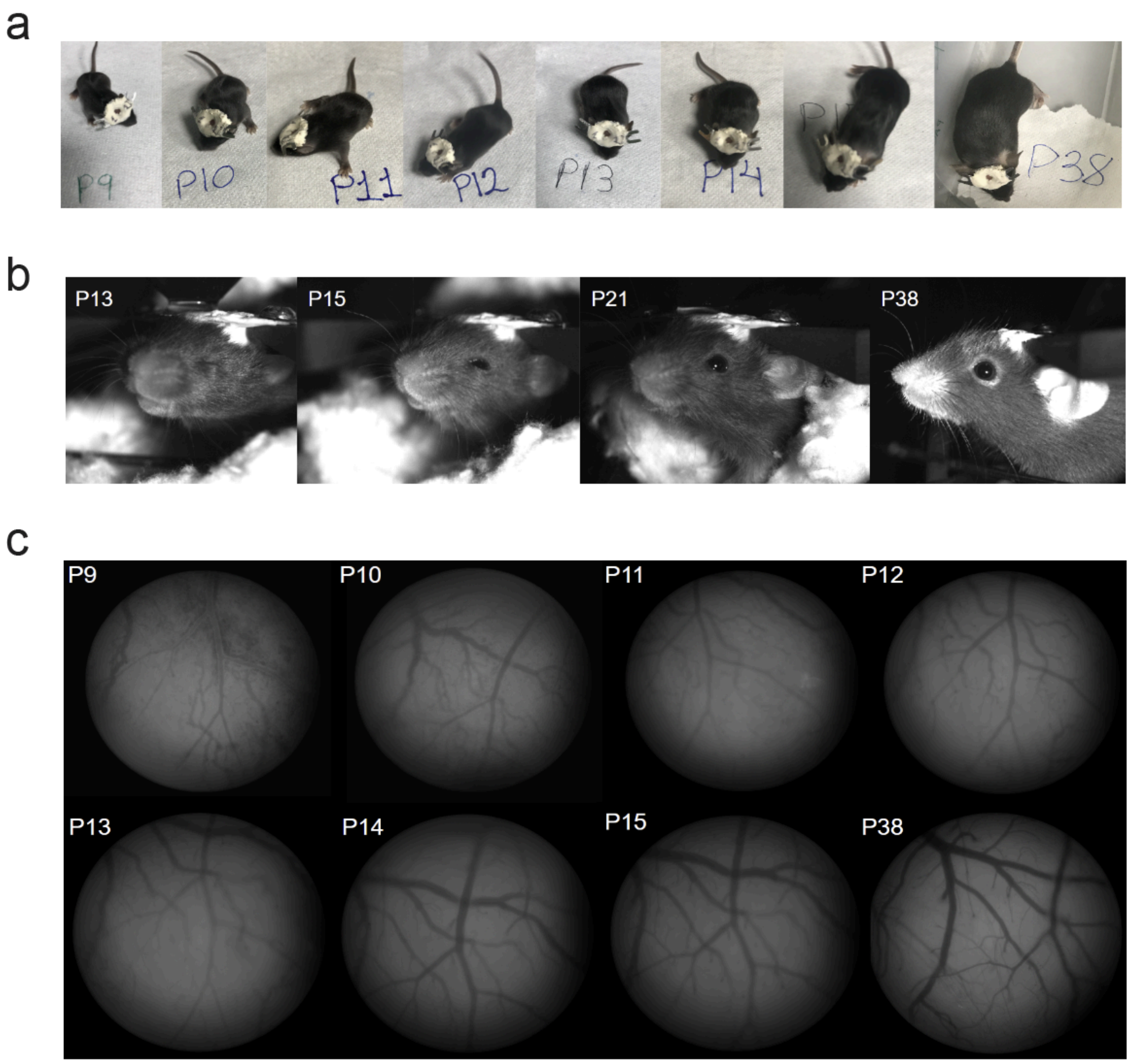


**Supplementary Fig. S9: Physiological development of mice with developmental implantation of a cranial window and a head-fixing plate. a,** Pictures of the same animal across development with the implants. **b,** Infrared pictures of the same animal head-fixed in different developmental stages, illustrating normal eye opening timeline. **c,** 1-photon low-magnification pictures of the craniotomy showing estable visibility without skull regrowth throughout development.

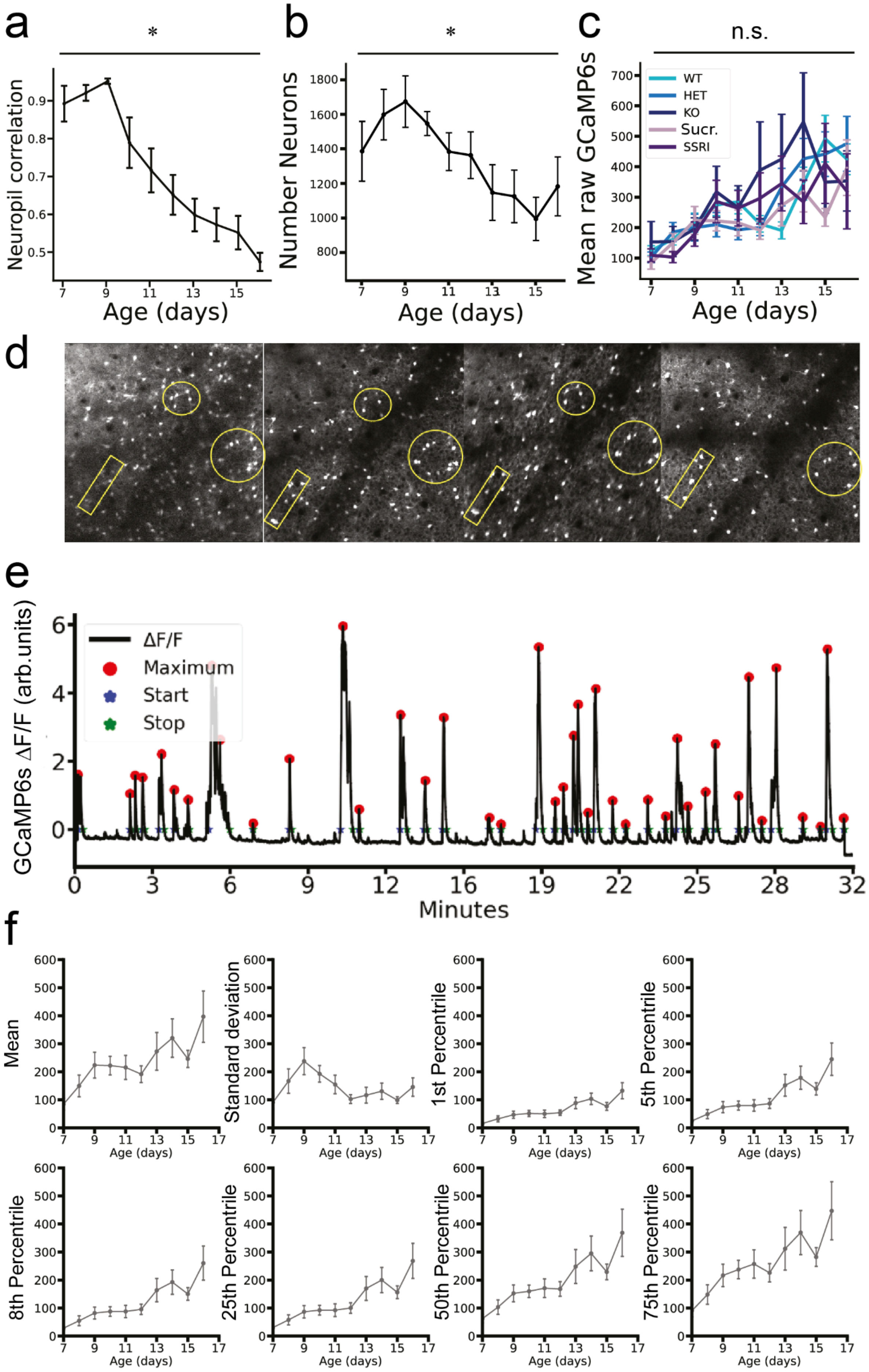

a
*
Neuropil correlation
Age (days)
b
*
Number Neurons
Age (days)
c
n.s.
Mean raw GCaMP6s
WT
HET
KO
Sucr.
SSRI
Age (days)
d
e
GCaMP6s ΔF/F (arb.units)
ΔF/F
Maximum
Start
Stop
Minutes
f
Mean
Standard deviation
1st Percentrile
5th Percentrile
8th Percentrile
25th Percentrile
50th Percentrile
75th Percentrile
Age (days)

**Supplementary Fig. S10. Developmental imaging of calcium traces from the same neurons throughout development. a,** Average neuropil-neuron Pearson's correlation coefficient during 20 minute baseline through development (P7-16) for all imaged animals. **b,** Average number of neurons detected in the field of view through development (P7-16) for all imaged animals. **c,** Mean raw calcium signal across development (P7-16) in SERT-WT/Het/KO and Sucrose-/SSRI- postnatally-treated mice (Two-way ANOVA: genotype $p = 0.491$, age $p < 0.001$, age-genotype $p < 0.001$). Sample sizes: n = 19 WT, n = 23 SERT-Het, n = 10 SERT-KO, n = 12 Sucrose and n = 12 SSRI mice. **d,** Tracking of the same interneurons (VIP+) across development (from left to right: P8, P10, P12 and P14). Images are averages of 128 frames used to locate the same FOV every imaging day. **e,** Representative average calcium trace of all cells in the field of view from a single animal, with the result of the calcium event automatic detection algorithm showing the maximum, start and end of each event, demonstrating high-accuracy to identify H- and L-events. **f,** Errorbar plots of the change of different statistics (mean, standard deviation, 1st, 5th, 8th, 25th, 50th and 75th percentile) in calcium dynamics, across development, to select the right normalisation approach for developmental recordings. Traces are the average of all imaged animals.

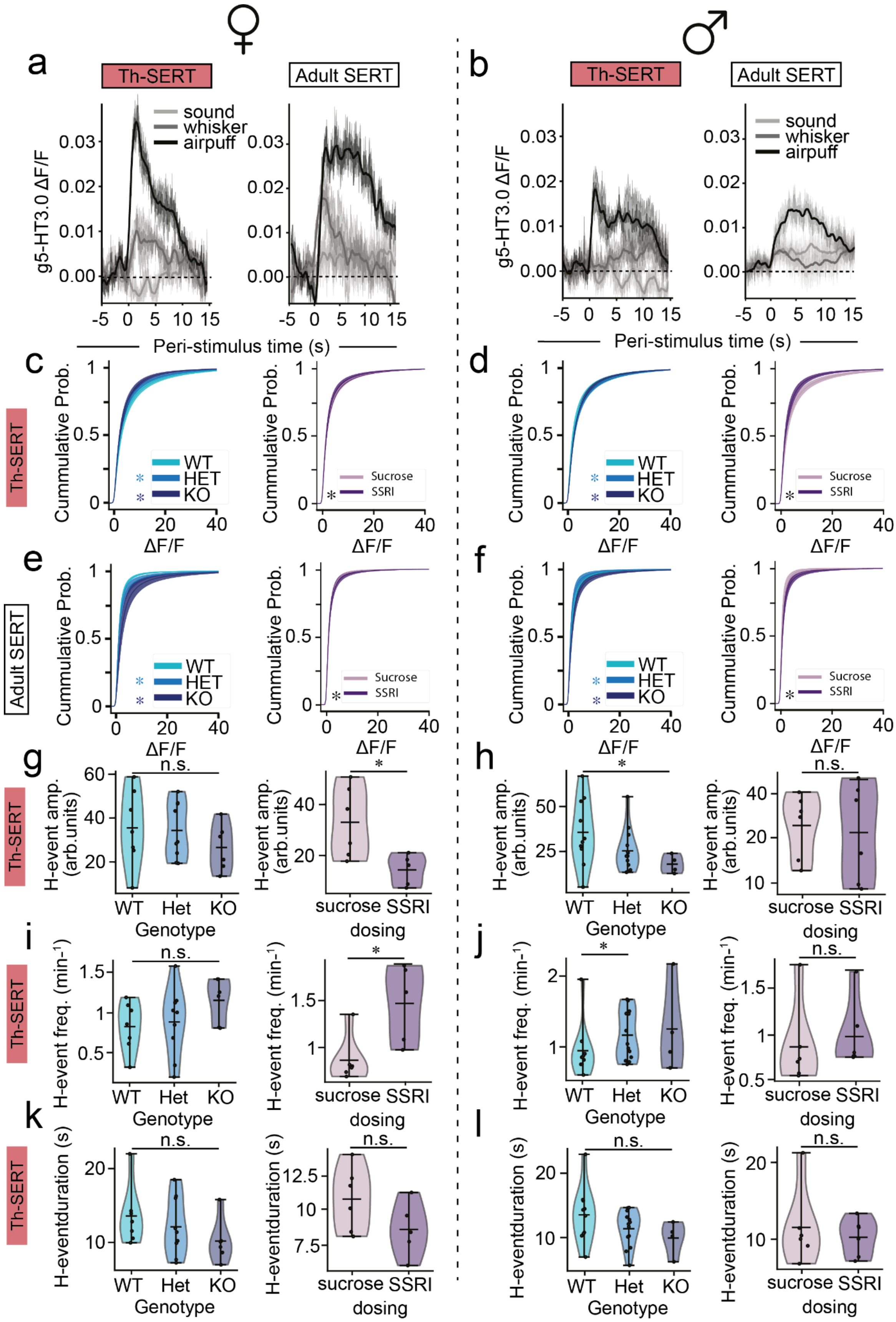
a
Th-SERT
Adult SERT
sound
whisker
airpuff
g5-HT3.0 ΔF/F
Peri-stimulus time (s)
b
c
d
e
f
Cummulative Prob.
WT
HET
KO
Sucrose
SSRI
ΔF/F
Adult SERT
g
h
H-event amp. (arb.units)
n.s.
Genotype
sucrose SSRI
dosing
i
j
H-event freq. (min-1)
k
l
H-eventduration (s)

**Supplementary Fig. S11. Female versus male serotonin and calcium dynamics in SERT-WT/Het/KO and Sucrose-/SSRI-treated mice. a,b,** Serotonin sensor g5-HT3.0 dynamics in response to airpuff, whisker or auditory stimuli during Th-SERT (**left**) and Adult SERT (**right**) periods in female (**a,** n = 2 mice) and male (**b,** n = 4) mice. **c-f** Cumulative probability of ΔF/F 20 minute baseline values at Th-SERT (top) and Adult SERT (bottom) in SERT-WT/Het/KO (left) and Sucrose/SSRI-treated (right) female (**c,e**) and male mice (**d,f**) (* $p < 0.05$). **g,i,k** Violin plots of average amplitude (**e**), frequency (**f**) and duration (**g**) of H-events in SERT-WT/HET/KO (Two-way ANOVA, **g,** genotype p = 0.811, **i,** genotype p = 0.265, and **k,** genotype p = 0.162) or Sucrose-/SSRI-treated mice (**g,** t-test p = 0.022; **i** Mann–Whitney U p = 0.017; **k,** t-test p = 0.131). **h,j,l** Violin plots of average amplitude (**e**), frequency (**f**) and duration (**g**) of H-events in SERT-WT/HET/KO (Two-way ANOVA, **h,** genotype p = 0.002, pairwise Dunn test WT-HET p = 0.009 and WT-KO p = 0.009; **j,** genotype p = 0.005, pairwise Dunn test WT-HET p = 0.013 and WT-KO p = 0.132; and **l,** genotype p = 0.084) or Sucrose-/SSRI-treated mice (**h,** t-test p = 0.775; **j** Mann–Whitney U p = 0.229; **l,** t-test p = 0.818).
