# Supplementary material for: Perinatal serotonin signalling dynamically influences the development of cortical GABAergic circuits with consequences for lifelong sensory encoding
Source: Nat Commun. 2025 Jun 4;16:5203. doi: 10.1038/s41467-025-59659-5 (PMC12137630; doi:10.1038/s41467-025-59659-5)
Supplement: Supplementary file 2 — Description of Additional Supplementary Files [file 41467_2025_59659_MOESM2_ESM.pdf]

Description of Additional Supplementary Files

File Name: Supplementary Movie 1.

Description: Serotonin sensor g5-HT3.0 signalling (average ΔF/F) following whisker stimulation in L2/3 S1BF of a P8 SERT-KO mouse (1x speed).

File Name: Supplementary Movie 2.

Description: Low synchronicity GCaMP6s calcium signal (L-) events recorded in S1BF (left panel) following whisker twitches during active sleep in a postnatal (P8) mouse (right panel).

File Name: Supplementary Movie 3.

Description: High synchronicity GCaMP6s calcium (H-) signal events recorded in S1BF (right panel) during awake in a postnatal (P8) mouse (right panel). Periods of awake were characterized by co-ordinated movements over a period of seconds
