# Supplementary material for: Perinatal serotonin signalling dynamically influences the development of cortical GABAergic circuits with consequences for lifelong sensory encoding
Source: Nat Commun. 2025 Jun 4;16:5203. doi: 10.1038/s41467-025-59659-5 (PMC12137630; doi:10.1038/s41467-025-59659-5)
Supplement: Supplementary file 6 — Reporting Summary [file 41467_2025_59659_MOESM6_ESM.pdf]

nature portfolio

Corresponding author(s): Simon J. B. Butt

Last updated by author(s): Mar 31, 2025

# Reporting Summary

Nature Portfolio wishes to improve the reproducibility of the work that we publish. This form provides structure for consistency and transparency in reporting. For further information on Nature Portfolio policies, see our Editorial Policies and the Editorial Policy Checklist.

## Statistics

For all statistical analyses, confirm that the following items are present in the figure legend, table legend, main text, or Methods section.

| n/a | Confirmed | |
|---|---|---|
| ☐ | ☒ | The exact sample size (*n*) for each experimental group/condition, given as a discrete number and unit of measurement |
| ☐ | ☒ | A statement on whether measurements were taken from distinct samples or whether the same sample was measured repeatedly |
| ☐ | ☒ | The statistical test(s) used AND whether they are one- or two-sided<br>*Only common tests should be described solely by name; describe more complex techniques in the Methods section.* |
| ☐ | ☒ | A description of all covariates tested |
| ☐ | ☒ | A description of any assumptions or corrections, such as tests of normality and adjustment for multiple comparisons |
| ☐ | ☒ | A full description of the statistical parameters including central tendency (e.g. means) or other basic estimates (e.g. regression coefficient) AND variation (e.g. standard deviation) or associated estimates of uncertainty (e.g. confidence intervals) |
| ☐ | ☒ | For null hypothesis testing, the test statistic (e.g. *F*, *t*, *r*) with confidence intervals, effect sizes, degrees of freedom and *P* value noted<br>*Give P values as exact values whenever suitable.* |
| ☒ | ☐ | For Bayesian analysis, information on the choice of priors and Markov chain Monte Carlo settings |
| ☒ | ☐ | For hierarchical and complex designs, identification of the appropriate level for tests and full reporting of outcomes |
| ☒ | ☐ | Estimates of effect sizes (e.g. Cohen's *d*, Pearson's *r*), indicating how they were calculated |

*Our web collection on statistics for biologists contains articles on many of the points above.*

## Software and code

Policy information about availability of computer code

| | |
|---|---|
| Data collection | Prairie View software (Bruker) was used for 2-photon image acquisition<br>Sensory stimuli were generated pseudo-randomly using custom MATLAB code (see Butt lab Github).<br>pClamp v10 (Axoscope, Clampex and Clampfit) were used for recording ex vivo ephys (all Molecular Devices, USA) |
| Data analysis | All 2-photon imaging data were pre-processed by registration with Turboreg.<br>Suite2p (v.0.14.0) was used to perform registration, cell detection and fluorescent trace extraction of all calcium recordings.<br>DeepLabCut (v2.3.5 and later) was used for analysis of all behavior.<br>Analysis of LSPS data was performed with a customized MATLAB (R2020a, v9.8 and later, MathWorks) toolbox (Butt lab Github)<br>Qupath (v0.5.0) was used for automated analysis of immunohistochemistry data |

For manuscripts utilizing custom algorithms or software that are central to the research but not yet described in published literature, software must be made available to editors and reviewers. We strongly encourage code deposition in a community repository (e.g. GitHub). See the Nature Portfolio guidelines for submitting code & software for further information.

## Data

Policy information about availability of data

All manuscripts must include a data availability statement. This statement should provide the following information, where applicable:

- Accession codes, unique identifiers, or web links for publicly available datasets
- A description of any restrictions on data availability
- For clinical datasets or third party data, please ensure that the statement adheres to our policy

A source data files has been provided. Full imaging and electrophysiology datasets are available by contacting the corresponding author. Summary data will be available via the University of Oxford open data archive: https://ora.ox.ac.uk/ with a hyperlink on the Butt lab website.

## Research involving human participants, their data, or biological material

Policy information about studies with human participants or human data. See also policy information about sex, gender (identity/presentation), and sexual orientation and race, ethnicity and racism.

| | |
|---|---|
| Reporting on sex and gender | N/A |
| Reporting on race, ethnicity, or other socially relevant groupings | N/A |
| Population characteristics | N/A |
| Recruitment | N/A |
| Ethics oversight | N/A |

Note that full information on the approval of the study protocol must also be provided in the manuscript.

# Field-specific reporting

Please select the one below that is the best fit for your research. If you are not sure, read the appropriate sections before making your selection.

☒ Life sciences ☐ Behavioural & social sciences ☐ Ecological, evolutionary & environmental sciences

For a reference copy of the document with all sections, see nature.com/documents/nr-reporting-summary-flat.pdf

# Life sciences study design

All studies must disclose on these points even when the disclosure is negative.

| | |
|---|---|
| Sample size | Sample sizes were not predetermined. All sample sizes are reported. |
| Data exclusions | No data points were excluded |
| Replication | This is not applicable. Multiple lines of evidence were used to support each conclusion. |
| Randomization | Groups were assigned post hoc based on genotype (determined post hoc) or treatment (e.g., SSRI vs. control). |
| Blinding | All experiments were performed blind to the sample genotype/treatment. Genotyping and treatment were performed by individuals not involved in the experiments. |

# Reporting for specific materials, systems and methods

We require information from authors about some types of materials, experimental systems and methods used in many studies. Here, indicate whether each material, system or method listed is relevant to your study. If you are not sure if a list item applies to your research, read the appropriate section before selecting a response.

## Materials & experimental systems

| n/a | Involved in the study |
| --- | --- |
| [ ] | [x] Antibodies |
| [x] | [ ] Eukaryotic cell lines |
| [x] | [ ] Palaeontology and archaeology |
| [ ] | [x] Animals and other organisms |
| [x] | [ ] Clinical data |
| [x] | [ ] Dual use research of concern |
| [x] | [ ] Plants |

## Methods

| n/a | Involved in the study |
| --- | --- |
| [x] | [ ] ChIP-seq |
| [x] | [ ] Flow cytometry |
| [x] | [ ] MRI-based neuroimaging |

# Antibodies

Antibodies used

antibody details are reported in the methods and repeated here:
rabbit anti-PV (PV27a, Swant, Switzerland; lot# AB2631173)
rabbit anti-SERT (Millipore Corp., USA; PC177L, lot# 3996287)
goat anti-Rabbit-Alexa568 (Invitrogen A11011, lots# 2273773;2782620)
goat anti-Rabbit-Alexa647 (Invitrogen A21245, lot# AB_2535813)

Validation

All antibodies are previously validated in peer review publications (e.g. anti-PV ab: Shelton et al. 2024, eLife13:RP98002). The anti-SERT was also tested on SERT-KO tissue as reported in the manuscript (Fig.S1c).

# Animals and other research organisms

Policy information about studies involving animals; ARRIVE guidelines recommended for reporting animal research, and Sex and Gender in Research

Laboratory animals

Mouse; all were maintained on C56BL/6J backgrounds. The following genetically modified lines were used:
SERT- mutant mice (Slc6a4(tm1Kpl/J))
Nkx2-1-Cre (Tg(Nkx2-1-cre)2Sand/J)
SST-ires-Cre (Ssttm2.1(cre)Zjh/J)
Ai32 (Gt(ROSA)26Sortm32 (CAG-COP4*H134R/EYFP)Hze/J)
Ai9 (Cg-Gt(ROSA)26Sortm9(CAG-tdTomato)Hze/J)
Olig3-Cre (Olig3tm1(cre)Ynka)
Dlx5/6-Cre (Tg(mI56i-cre,EGFP)1Kc/J)
conditional Celsr3 (Celsr3fl/fl)

Wild animals

No wild animals were used

Reporting on sex

Mice of either sex were used throughout.

Field-collected samples

N/A

Ethics oversight

Experiments were approved by the local ethical review committee at the University of Oxford and performed in accordance with UK Home Office project licenses P861F9BB7, PE5B24716 and PP8136190 under the UK Animals (Scientific Procedures) 1986 Act.

Note that full information on the approval of the study protocol must also be provided in the manuscript.

# Plants

Seed stocks

N/A

Novel plant genotypes

N/A

Authentication

N/A



April 2023
